# Freezing point temperature is in favor of not‐from‐concentrate apple juice storage

**DOI:** 10.1002/fsn3.1028

**Published:** 2019-05-29

**Authors:** Xiaoju Gou, You Tian, Xi Yang, Lijun Sun, Yurong Guo

**Affiliations:** ^1^ College of Food Engineering and Nutritional Science Shaanxi Normal University Xi'an China

**Keywords:** apple juice, freezing point temperature, multivariate statistical analysis, not from concentrate

## Abstract

The short storage period is still a problem hindering the promotion of not‐from‐concentrate (NFC) apple juice, despite the fact it possesses higher nutritional value and more attractive taste compared with its concentrated counterparts. In this study, we compared the effects of temperature range including room temperature (25°C), refrigerator temperature (4°C), freezing point temperature (−1.5°C), and frozen temperature (−18°C), respectively, on the quality of NFC apple juices during a long storage period (150 days). The results suggested that all the juices exhibited good safety during the storage, and the juice stored at −1.5°C possessed higher polyphenol contents, physicochemical properties, less color alteration, and less loss of aroma and taste than 25 and 4°C. Besides, although an exceedingly low temperature (−18°C) could greatly retard the juice deterioration, the loss of aroma and taste was significant. Overall, our results indicated that the NFC juice was most favored by storage at freezing point temperature (−1.5°C), with the highest similarity to the freshly squeezed apple juice.

## 1 INTRODUCTION

Apple is one of the most important fruits consumed around the world, and the annual consumption was reported up to 64.6 million tons (Mditshwa, Fawole, & Opara, 2018). Apple juice may provide an alternative to fresh fruit due to its pleasant taste and high nutritional values (Zhang et al., 2018). European Union (EU) legislation has defined two categories of fruit juice: “fruit juice” and “fruit juice reconstituted from concentrate,” which are described as not from concentrate (NFC) and from concentrate (FC), respectively (Wodarska, Khmelinskii, & Sikorska, 2018). The NFC apple juice, obtained directly from fruit, is increasingly recognized as a premium market segment (Tian, Gou, Niu, Sun, & Guo, 2018). As an example, according to the European Fruit Juice Association Liquid Fruit Market Report (2015), the consumption of NFC juice has been reported to increase over the last 5 years in the EU, while the consumption of FC juice was decreased.

Currently, it has been proven that fruit juice stored at room temperature has a significantly shorter shelf life than that stored at a lower temperature (Teleszko, Nowicka, & Wojdyo, 2016). However, it has been reported that when the juice was stored at a low temperature (i.e., 4°C), the polyphenol content was decreased (Tembo, Holmes, & Marshall, 2017). Besides, as the temperature was further decreased (i.e., −18°C), the safety and shelf life of the juice could be largely improved (Archer, 2004), whereas the frozen juice requires to be thawed before further processing, during which the juice may face with the possible secondary infection of microorganisms. As a result, the appropriate storage temperature is a perquisite for maintaining juice qualities. Freezing point temperature, slightly higher than supercooling point, has been used for the storage of animal organs and fresh fish (Wang et al., 2018; Zhu, Ma, Yang, Xiao, & Xiong, 2016). It has been reported that the nectarine stored at freezing point (−1.5°C) exhibited preferably physiological qualities and better antioxidant capacity than at 0 or 5°C (Zhao, Shu, Fan, Cao, & Jiang, 2018). Also, storing green beans at near its freezing point has been proven to retard the deterioration of the qualities of the beans (Guo, Ma, Sun, & Wang, 2008).

By far, it has been well established that the freezing point temperature can exert a more significant effect on the maintenance of the qualities of fruits. However, the reports concerning the temperature effects on NFC apple juice quality are very limited. Thus, this study was used to evaluate the effects of the storage temperature on the quality of NFC apple juices, including room temperature (25°C), low temperature (4°C), freezing point temperature (determined to be around −1.5°C) and frozen temperature (−18°C). Additionally, hierarchical cluster analysis (HCA) was applied to pinpoint the difference between the stored NFC apple juice and the freshly squeezed apple juice based on their polyphenolic contents. Furthermore, principal component analysis (PCA) and linear discriminant analysis (LDA) were used to distinguish the juices so as to emphasize their differences in taste.

## 2 MATERIALS AND METHODS

### 2.1 Juice preparation and storage

Fresh “Fuji” apples were purchased from a local market (Xi'an, China). The apples were washed, peeled, and then the seeds and cores were also removed, after which the apple flesh was cut into slices (6 mm in diameter and 5 mm in thickness). Immediately, the slices were immersed into 0.6% ascorbic acid solution to avoid the enzymatic browning. Afterward, the slices were squeezed by using a juice extractor (HU‐780WN, Hurom, Korea) and the juice was collected and then filtered with a 100‐mesh filter. After sterilization at 98°C for 30 s and canning, the juice was cooled to room temperature for further storage. Four temperatures including room temperature (25°C), refrigerator temperature (4°C), freezing point temperature, and frozen temperature (−18°C) were selected to store the juice. For each temperature, physicochemical properties and microorganisms of the juice were investigated after a time interval of 30 days. Besides, the polyphenol content, aroma, and taste were also determined after 150 days of storage.

### 2.2 Measurement of freezing point temperature

The freezing point temperature was measured according to a reported method by Jie, Lite, Lite, and Yang (2003) with some modifications (Jie et al., 2003). When the juice was cooled at a rate of 0.5°C/min, the temperature of the juice was recorded per 2 s using a digital thermometer (testo 106, Testo, Germany) and plotted as a function versus temperature. The corresponding temperature plateau at which the juice did not exhibit discernible temperature change was defined as the freezing point temperature of the juice.

### 2.3 Detection of microorganisms

Aerobic bacterial counts were determined according to the national standard (GB 4789.2‐2016) in China. After plates were incubated at 36°C for 48 hr, the amounts of microorganic colonies were recorded. Besides, yeasts and molds were also detected according to the method defined by the national standard (GB 4789.15‐2016) in China. Briefly, 1 ml of the 10‐fold diluted apple juice was inoculated into the Rose Bengal Agar (RBA) medium. After RBA plates were incubated at 28°C for 5 days, the amounts of yeast and mold colonies were calculated. The results were expressed as log (CFU/ml). According to the national standard (GB 4789.3‐2016) in China, coliforms were determined as follows: Juice samples were inoculated into the lauryl sulfate tryptose (LST) broth tubes, and subsequently, the matrixes were cultivated at 28°C for 48 hr to check whether there appeared the bubbles in the broth. Generally, the generation of the bubbles can be deemed to be indicative of coliforms.

### 2.4 Detection of physicochemical properties

The pH value was measured using a digital pH meter (FE20 Plus, Mettler‐Toledo, China). The titratable acidity (TA) was determined by using 0.1 M NaOH solution to titrate the 20‐fold diluted juice, and phenolphthalein served as the pH indicator. The results were expressed as g/L, with malic acid as the reference. The total soluble solid (TSS) was measured using a refractometer (PAL‐1, Atago, Japan), and the result was expressed as °Brix. Besides, the TSS/TA is calculated as the ratio of TSS content to TA content. The turbidity was determined using a turbidimeter (ET76910, Lovibond, Germany). Before testing, all the juices were diluted 10‐fold with distilled water.

Based on the CIE color system, the color values of the juices were measured using a colorimeter (NS800, 3nh, China). The L*, a*, and b* values correspond to lightness, greenness (−a*) or redness (+a^*^), and blueness (−b*) or yellowness (+b*), respectively. The total color difference (Δ*E*) was calculated using the following equation:

(1) $$\Delta E={\left[{\left(L^{*}-L_{0}^{*}\right)}^{2}+{\left(a^{*}-a_{0}^{*}\right)}^{2}+{\left(b^{*}-b_{0}^{*}\right)}^{2}\right]}^{0.5}$$

### 2.5 UPLC analysis of polyphenolic compositions

The mixture of the apple juice (3 ml) and 80% methanol (6 ml, *v*/*v*) was sonicated at 25°C for 15 min, subsequently centrifuged at 7,104 *g* at 4°C for 10 min. The supernatant was collected and then filtrated through a 0.22‐μm Teflon membrane for UPLC analysis. The qualification and quantification analysis of polyphenols was performed according to our previous method (Sun et al., 2017) using a UPLC system equipped with a Thermo^®^ Syncronis C18 column (250 × 4.6 mm, 5 μm, I.D., USA) and a Thermo^®^ Ultimate 3000 UPLC system (Thermo Electron Co. USA). The eluant consisted of A (30% acetonitrile and 70% methanol) and B (1‰ trifluoroacetic acid and 5% methanol). The elution was performed as follows: 0–3 min, 100% B at a flow rate of 0.95 ml/min; 3–19.05 min, 100%–60% B at a flow rate of 0.95 ml/min; 19.05–30.1 min, 60% B at a linear increasing flow rate ranging from 0.95 to 1 ml/min; 30.1–30.2 min, 60%–100% B. The detection wavelength was 280 nm, and the injection volume was 4 μl.

### 2.6 Electronic nose analysis

The aroma of the NFC juices was analyzed using an electronic nose (Smartnose, ISENSO, China) containing 10 metal‐oxide semiconductor chemical sensors. The detailed information is given in Table S1. Prior to analysis, the gas path of e‐nose was purged by clean air for 30 min to normalize the sensor signals. After that, 10 ml of the apple juice was transferred into a 50‐ml glass vial equipped with a plastic septum and the juice was equilibrated for 5 min to ensure the volatiles were fully collected. Then, the volatiles lingering in headspace were absorbed by a Luer‐lock needle connected to a Teflon tubing (3 mm) at a flow rate of 300 ml/min. The data were collected at a time interval of 0.1 s. The response of sensor was expressed as the ratio of conductance G/G_0_ (G_0_ and G represent the conductance of sensor before and after being exposed to the gas samples, respectively).

### 2.7 Electronic tongue analysis

An electronic tongue (Smartongue, ISENSO, China) composed of six metallic disk electrodes (platinum, gold, palladium, titanium, tungsten, and silver) was applied for the taste analysis of the NFC juices. Prior to detection, the electronic tongue (e‐tongue) was warmed up for 30 min using 0.01 M KCl solution and the metallic sensors were subsequently washed with distilled water for 2–3 min, so as to minimize the nose interference. When analyzed, the juices were filtrated with a filtrating membrane to avoid the artifact signals caused by the solid particles and the temperature was 25°C.

### 2.8 Statistical analysis

SPSS 18.0 software (SPSS Inc., USA) was used to analyze the obtained data, and the results were expressed as the means ± standard deviations. The significant difference was calculated using Duncan's test at a level of *p* < 0.05. Besides, PCA, LDA, and HCA were also performed to pinpoint the difference between the NFC apple juices.

## 3 RESULTS AND DISCUSSION

### 3.1 Freezing point temperature of NFC apple juice

The temperature curve of the NFC juice is displayed in Figure S1. As expected, the temperature of the juice was observed to decrease to its supercooling point temperature (−1.8°C), and then, the temperature was slightly increased and formed a distinct plateau. Thus, the temperature of the juice at the plateau region (−1.5°C) was regarded as the freezing point (Zhao et al., 2018).

### 3.2 Detection of microorganisms in NFC juices

**Figure 1:**
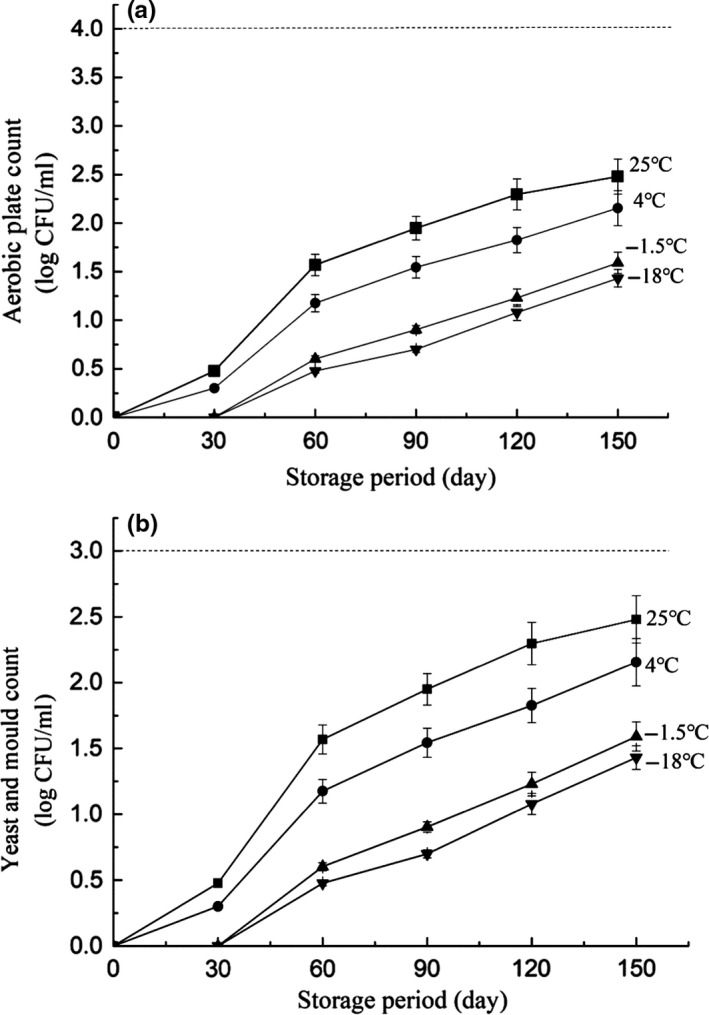
Aerobic plate count (a), and mold and yeast (b) of NFC juices during storage at different temperatures

The microorganism amounts of the juices during storage were regularly detected to reflect the effects of temperature on the juice safety. No aerobic bacterial counts, and yeast and mold counts were observed in the initial NFC juice, as reflected in Figure 1a,b. Regarding the juice stored at 25°C, both the aerobic plate counts (APC) and the yeast and mold counts (YMC) showed higher growing rates than at other storage temperatures. In detail, after 150 days of storage, the counts of APC and YMC were increased to 2.55 and 2.48 log CFU/ml, respectively. According to the Institute of Food Science and Technology, IFST (1999), the maximum acceptable microbial load regarding APC and YMC in fruit juices is 4 and 3 log CFU/ml, respectively. Thus, the juices studied in this work are of good safety. Besides, no coliform was observed during the storage (data not shown), confirming the safety of the juices.

Moreover, it was also found that the microbial loads of APC and YMC were decreased in parallel with the decreased temperatures. Specially, the juice stored at −18°C showed the lowest microorganism count, and the APC and YMC loads were 1.52 and 1.41 log CFU/ml after 150 days of storage, respectively. It is worth noting that laboratory production may be different from industrial scale, which may result from the fact that the greater loads in industrial production could cause the secondary infections of microorganisms. For example, in laboratory tests, approximately 50 ml of juices was used for quality analysis, whereas on industrial scale, a batch juice of around 220 L is normally required. That may cause a significant longer thawing process during which the microorganism infection possibly occurs.

### 3.3 Physicochemical properties of NFC juices

During the storages at different temperatures, the pH values were observed to be decreased, and the TA contents were increased, which may be due to the acidic metabolites produced by yeasts and lactobacillus. As discussed above, low temperature can inhibit the growth of microorganisms, and thus, the juice stored at −18°C showed the lowest fluctuations of pH and TA. Moreover, it can be seen that the TSS of juices was significantly increased during the early storage period and then followed by a decreasing trend (Table 1A). Notably, the juice stored at −1.5 and −18°C displayed a delayed increase in TSS compared with the higher temperature (i.e., 4 and 25°C), indicating that the maintenance of TSS can be favored by a lower temperature. Additionally, the ratio of TSS to TA is usually calculated to assess the overall taste of juice because the TSS/TA ratio has been reported to have a stronger correlation to the taste of juice than TSS and/or TA alone (Crisosto & Crisosto, 2005). As listed in Table 1A, the TSS/TA ratio of the juices was significantly decreased after 150 days of storage, suggesting that the juice was characterized by a more acidic taste. The result may indicate that the acceptability of the juice was decreased, because the previous works have demonstrated that a higher TSS/TA ratio is commonly associated with higher juice acceptability (Ortiz, Echeverría, Graell, & Lara, 2009; Ortiz, Graell, López, Echeverría, & Lara, 2010). However, low temperatures can facilitate the retention of the TSS/TA ratio. After 150 days of storage, the TSS/TA ratio in the juice at −18°C was about 30, significantly higher than other temperatures.

**Table 1:** Physicochemical properties (A) and color values (B) of juices stored at different temperatures

(A)		pH	Titratable acid (g/L)	Total soluble solids (°Brix)	TSS/TA	Turbidity (NTU)
	0 day	3.73 ± 0.01^a^	3.74 ± 0.11^k^	13.22 ± 0.16^g^	35.36 ± 1.09^ab^	2,363.33 ± 28.67^a^
25°C	30 days	3.71 ± 0.01^ab^	3.92 ± 0.18^ij^	13.63 ± 0.05^cde^	34.81 ± 1.73^bc^	2,126.67 ± 4.71^defg^
	60 days	3.66 ± 0.01^cd^	4.43 ± 0.07^f^	13.77 ± 0.05^abc^	31.11 ± 0.42^de^	1,960.21 ± 8.16^h^
	90 days	3.44 ± 0.02^g^	4.72 ± 0.03d^e^	13.13 ± 0.09^gh^	27.85 ± 0.11^f^	1,826.67 ± 4.71^jk^
	120 days	3.02 ± 0.01^j^	4.87 ± 0.07^bc^	12.51 ± 0.08^j^	25.66 ± 0.34^g^	1,760.43 ± 8.16^l^
	150 days	2.93 ± 0.04^k^	5.33 ± 0.06^a^	12.33 ± 0.05^k^	23.29 ± 0.24^h^	1,606.67 ± 24.94^m^
4°C	30 days	3.72 ± 0.01^ab^	3.86 ± 0.07^jk^	13.57 ± 0.05^de^	35.18 ± 0.53^ab^	2,190.21 ± 12.45^bcd^
	60 days	3.69 ± 0.01^bc^	4.09 ± 0.07^gh^	13.83 ± 0.09^ab^	33.81 ± 0.41^c^	2,103.33 ± 106.25^efg^
	90 days	3.52 ± 0.01^f^	4.32 ± 0.05^f^	13.63 ± 0.05^cde^	31.61 ± 0.31^de^	1,910.11 ± 11.45^hi^
	120 days	3.22 ± 0.02^i^	4.75 ± 0.11^cd^	13.03 ± 0.05^h^	27.45 ± 0.51^f^	1,856.67 ± 47.84^ij^
	150 days	3.03 ± 0.02^j^	4.91 ± 0.04^b^	12.83 ± 0.05^i^	26.16 ± 0.32^g^	1,776.67 ± 4.71^kl^
−1.5°C	30 days	3.71 ± 0.01^ab^	3.76 ± 0.04^k^	13.62 ± 0.08^de^	36.21 ± 0.39^a^	2,243.33±17^b^
	60 days	3.69 ± 0.01^bc^	4.06 ± 0.06^ghi^	13.71 ± 0.02^bcd^	33.76 ± 0.52^c^	2,196.67 ± 4.71^bc^
	90 days	3.64 ± 0.02^d^	4.11 ± 0.05^g^	13.87 ± 0.05^a^	33.71 ± 0.55^c^	2,086.67 ± 4.71^fg^
	120 days	3.53 ± 0.01^f^	4.39 ± 0.08^f^	13.53 ± 0.05^e^	30.82 ± 0.58^de^	1,940.67 ± 37.42^h^
	150 days	3.42 ± 0.02^h^	4.59 ± 0.06^e^	13.37 ± 0.05^gh^	28.67 ± 0.42^f^	1,806.67 ± 20.55^jkl^
−18°C	30 days	3.72 ± 0.01^ab^	3.76 ± 0.04^k^	13.62 ± 0.08^de^	36.21 ± 0.61^a^	2,240.32 ± 29.44^b^
	60 days	3.69 ± 0.01^bc^	3.96 ± 0.04^hij^	13.77 ± 0.05^abc^	34.78 ± 0.36^c^	2,150.67 ± 8.16^cdef^
	90 days	3.67 ± 0.02^cd^	4.15 ± 0.05^g^	13.91 ± 0.03^a^	33.52 ± 0.44^c^	2,163.33 ± 18.86^cde^
	120 days	3.61 ± 0.02^e^	4.34 ± 0.06^f^	13.63 ± 0.05^cde^	31.44 ± 0.45^de^	2,133.33 ± 23.57^defg^
	150 days	3.53 ± 0.01^f^	4.44 ± 0.04^f^	13.39 ± 0.05^f^	30.12 ± 0.37^e^	2,080.23 ± 14.14^g^

(B)		L*	a*	b*	Δ*E*	
	0 day	30.67 ± 0.23^efg^	−2.98 ± 0.059^hij^	3.52 ± 0.85^ab^	—	
25°C	30 days	31.21 ± 0.12^cdef^	−3.13 ± 0.1^j^	4.18 ± 1.4^a^	0.87	
	60 days	32.13 ± 0.01^bc^	−2.71 ± 0.03^cdefg^	0.61 ± 0.05^cde^	3.27	
	90 days	29.65 ± 0.38^hi^	−2.46 ± 0.11^abc^	1.37 ± 1.26^bcde^	2.49	
	120 days	29.40 ± 0.93^hi^	−2.49 ± 0.19^abcd^	0.32 ± 1.38^de^	3.47	
	150 days	27.45 ± 0.61^k^	−2.25 ± 0.18^a^	0.96 ± 1.54^e^	5.57	
4°C	30 days	31.54 ± 0.64^cde^	−3.12 ± 0.12^ij^	3.43 ± 0.23^ab^	0.89	
	60 days	32.97 ± 0.03^b^	−3.79 ± 0.02^k^	3.6 ± 0.16^ab^	1.85	
	90 days	30.74 ± 0.31^defg^	−2.76 ± 0.08^efgh^	1.68 ± 0.9^abcd^	2.44	
	120 days	30.27 ± 0.21^fgh^	−2.38 ± 0.1^ab^	0.04 ± 1.22^de^	3.56	
	150 days	29.62 ± 0.8^hi^	−2.58 ± 0.22^bcde^	0.63 ± 1.88^cde^	5.10	
−1.5°C	30 days	30.78 ± 0.06^defg^	−2.73 ± 0.04^defg^	2.33 ± 0.65^abcd^	1.23	
	60 days	31.68 ± 0.41^cd^	−2.88 ± 0.22^ghi^	3.49 ± 0.86^ab^	1.02	
	90 days	36.75 ± 0.04^a^	−2.73 ± 0.02^defg^	0.26 ± 0.09^de^	3.20	
	120 days	31.42 ± 0.5^j^	−2.24 ± 0.04^a^	0.03 ± 1.32^de^	4.27	
	150 days	31.80 ± 0.7ij	−2.37 ± 0.15ab	0.19 ± 1.33de	4.90	
−18°C	30 days	30.83 ± 0.08^defg^	−2.78±0^efgh^	2.09 ± 0.27^abcd^	1.45	
	60 days	31.01 ± 0.05^def^	−2.81 ± 0.03^fgh^	3.00 ± 0.73^abc^	1.48	
	90 days	29.96 ± 0.41^gh^	−2.68 ± 0.12^cdef^	1.65 ± 1.92^abcd^	2.03	
	120 days	31.56 ± 0.14^cde^	−2.58 ± 0.06^bcde^	3.45 ± 0.62^ab^	2.99	
	150 days	31.31 ± 0.04^cde^	−2.52 ± 0.09^bcde^	3.14 ± 0.09^f^	3.71	

Note

Values were expressed as means ± standard deviations. Values followed by different letters in the same column are significantly different (Duncan's test, *p* < 0.05).

Turbidity is another crucial parameter that represents the level of the cloudiness or haziness caused by the suspended solid particles (Cerreti, Liburdi, Benucci, & Esti, 2016); a higher turbidity means a higher content of flesh particles (Tian et al., 2018). In UFC juices, the flesh particles were mainly composed of proteins, cellulose, hemicellulose, and pectin. It can be found that the turbidity was continuously deceased during the storage. And the decrease in temperature could facilitate the retention of turbidity, as indicated by the higher turbidity in the juice stored at −1.5°C. That can be explained by the fact that the viscosity of the juice was higher at a lower temperature, which may reduce the precipitation rate of the suspended flesh particles. As expected, the juice stored at −18°C exhibited the greatest turbidity, which may be because the frozen juice immobilized the flesh particles. Thus, the precipitation of the particles was largely inhibited.

### 3.4 Color quality of NFC juices

Compared with the concentrated apple juice, NFC juice usually exhibits a color more close to the flesh of fresh apples, owing to the omitted concentration process during which the browning of the juice inevitably occurs. However, during a long term of storage, the color of NFC juice may be deepened due to the enzymatic browning. For example, it has been reported that both the oxidation of polyphenolic compounds and Maillard reaction can lead to the deepened color of the juice (Lee, Seo, Rhee, & Kim, 2016). In this study, L*, a*, b*, and Δ*E* values were applied to fully characterize the juice color, and the results are summarized in Table 1B. As listed in the table, the L*, a*, and b* values of all the juices were found to be continuously decreased during the whole storage period, suggesting that the juices had a deeper color as the storage proceeded. In addition, it can be found that the total color difference (*∆E*), which is generally used to characterize the comprehensive color difference between the test group and the control group, was continuously increased. The result suggested that the color of the stored juices exhibited a widened difference as the storage was prolonged. Moreover, the juice color could be better maintained as the storage temperatures decreased, which is in agreement with a previous study reporting that the decrease in storage temperature was associated with a lower Δ*E* value (Buvé et al., 2018). Generally, when Δ*E* value is ranged from 3.0 to 6.0, the color difference can be discerned by unaided eye (Wibowo et al., 2015). In our study, the Δ*E* values of all the juices were over 3.0 after 150 days of storage, indicating that the changes in color would be visible.

### 3.5 Qualification and quantification of polyphenols in NFC juices

**Figure 2:**
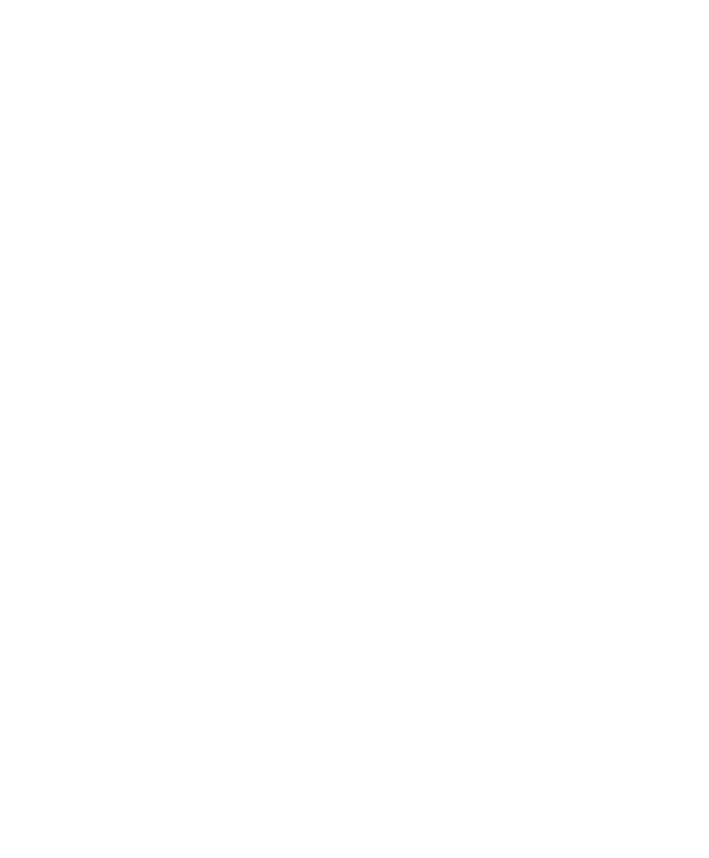
UPLC chromatogram of 14 individual polyphenols identified in NFC juices (a) and heatmap based on the polyphenol compositions (b)

UPLC was used to identify and quantify the individual phenolics in the NFC juices. Based on the peaks, fourteen polyphenolic compounds including thirteen known polyphenols and one unknown compound were identified, and the result is given in Table 2. Furthermore, 13 proposed individual phenolics were quantified by UPLC, and the results are displayed in Figure 2a. As can be seen in Figure 2a, the juices stored at different temperatures exhibited similar polyphenolic profiles but varied peak areas, suggesting that the composition of the individual polyphenols was the same but the polyphenol content was different. The further HCA and the yielded heatmap suggested that the stored juices can be divided into two groups: The juices stored at 25 and 4°C exhibited similar individual polyphenol contents and therefore were categorized into one group; the juices stored at −1.5 and −18°C showed high similarity of individual polyphenol contents and can be categorized into another group, as reflected in Figure 2b. However, there is significant difference observed between the two groups and the control (the freshly squeezed juice). The detailed contents of individual polyphenols are summarized in Table 2. It can be observed that the major phenolic compounds were 3‐caffeoylquinic acid, procyanidin B2, and epicatechin, which is consistent with a previous study (Heinmaa et al., 2017). Among the 13 identified individual polyphenols, epigallocatechin, catechin, procyanidin B2, epicatechin, and epicatechin gallate belong to procyanidins, and gallic acid, protocatechuic acid, chlorogenic acid, and 4‐hydroxybenzoic acid belong to the family of phenolic acids (Tian et al., 2018). It has been reported that the most abundant polyphenols in apples are procyanidins, followed by phenolic acids (Renard et al., 2011). In the NFC juice, however, chlorogenic acid (3‐caffeoylquinic acid) was found to account for the highest content of polyphenols, as listed in Table 2. It may be because the removal of peels during the NFC preparation greatly reduced the procyanidins and phloridzin contents, which mostly exist in apple peels (Chen, Zhang, Wang, Li, & Ma, 2012; Heinmaa et al., 2017). By contrast, chlorogenic acid is most abundant in apple flesh (Zhao et al., 2015). Moreover, it was found that the contents of individual polyphenols were roughly decreased during the storage period, suggesting that the polyphenols in the juices may be slightly degraded. Also, the increased storage temperature caused more pronounced polyphenol degradation, which is in line with the reports by Kim et al. (2018) and Teleszko et al. (2016). Due to significant antioxidant capacity, plant polyphenols are often involved into the free radical scavenging reaction to reduce the oxidative stress of organisms, and thus, the polyphenol content is commonly regarded as one crucial parameter for assessing the nutritional value of juices. At low temperatures (i.e., −1.5 and −18°C), the polyphenol degradation could be retarded and therefore the polyphenol contents were well maintained after 150 days of storage. However, the difference in molecular architecture allows the individual polyphenols to exhibit different stability. As an example, when the juice was stored at 25°C, the contents of epigallocatechin, procyanidin B2, epicatechin, and phloridzin exhibited a larger extent of decrease, as reflected in Table 2, demonstrating the instability of procyanidin (Dai et al., 2018). On the contrary, the contents of 3‐caffeoylquinic acid and protocatechuic acid were relatively stable during the whole storage, which is in accordance with a previous study (Rekas, Scibisz, Siger, & Wroniak, 2017).

**Table 2:** Retention time (Rt), wavelengths of maximum absorption (λmax), tentative identification, and quantification of the phenolic compounds of juices stored at different temperatures

Peak	Rt (min)	λmax	Tentative identification	CK (μg/ml)	25°C (μg/ml)	4°C (μg/ml)	−1.5°C (μg/ml)	−18°C (μg/ml)
1	9.5	275	Unknown	—	—	—	—	—
2	10.2	260	Protocatechuic acid	3.69 ± 0.17^a^	3.54 ± 0.23^a^	3.57 ± 0.09^a^	3.72 ± 0.87^a^	3.84 ± 0.98^a^
3	12.7	289	Procyanidin B1	11.01 ± 0.25^a^	8.94 ± 1.02^b^	9.27 ± 1.11^b^	9.06 ± 1.29^b^	11.64 ± 0.93^a^
4	13.2	280	Epigallocatechin	7.53 ± 0.97^ab^	6.33 ± 0.42^c^	6.84 ± 0.69^b^	5.39 ± 1.21^a^	5.41 ± 0.32^a^
5	13.5	279	Catechin	8.61 ± 1.21^ab^	6.81 ± 0.99^c^	8.91 ± 1.43^ab^	9.24 ± 1.32^a^	7.62 ± 0.93^b^
6	13.9	279	Procyanidin B2	58.59 ± 0.98^a^	49.23 ± 2.19^c^	52.14 ± 1.51^b^	53.46 ± 0.99^b^	55.89 ± 2.81^a^
7	14.4	325	3‐caffeoylquinic acid	166.83 ± 3.46^a^	163.17 ± 4.32^ab^	164.43 ± 4.31^ab^	166.26 ± 5.92^a^	167.07 ± 3.82^a^
8	14.9	311	*4‐p‐*coumaroylquinic acid	14.07 ± 2.01^a^	10.74 ± 1.03^b^	12.36 ± 1.02^ab^	13.56 ± 0.22^a^	13.95 ± 0.93^a^
9	15.4	279	Epicatechin	53.58 ± 0.78^a^	37.11 ± 1.23^c^	37.98 ± 2.01^c^	46.35 ± 0.94^b^	50.49 ± 0.28^a^
10	16.2	280	Procyanidin C1	9.06 ± 1.22^a^	4.47 ± 0.93^c^	5.13 ± 0.93^bc^	6.75 ± 0.32^b^	8.91 ± 0.97^a^
11	17.7	350	Quercetin‐3‐O‐glucoside	0.69 ± 0.04^a^	0.57 ± 0.03^b^	0.57 ± 0.03^ab^	0.63 ± 0.03^ab^	0.66 ± 0.01^a^
12	18.1	350	Quercetin‐3‐O‐arabinoside	3.69 ± 0.17^a^	3.54 ± 0.23^a^	3.57 ± 0.09^a^	3.72 ± 0.87^a^	3.84 ± 0.98^a^
13	19	350	Quercetin‐3‐O‐galactoside	6.93 ± 0.16^a^	5.63 ± 0.15^b^	5.76 ± 0.21^b^	6.65 ± 0.32^a^	6.97 ± 0.31^a^
14	20.3	285	Phlorizin	12.43 ± 2.28^a^	11.12 ± 1.47^b^	11.43 ± 2.75^b^	12.29 ± 1.56^a^	12.25 ± 1.73^a^

Note

Values were expressed as means ± standard deviations. Values followed by different letters in the same row are significantly different (Duncan's test, *p* < 0.05).

### 3.6 E‐nose analysis of NFC juices

**Figure 3:**
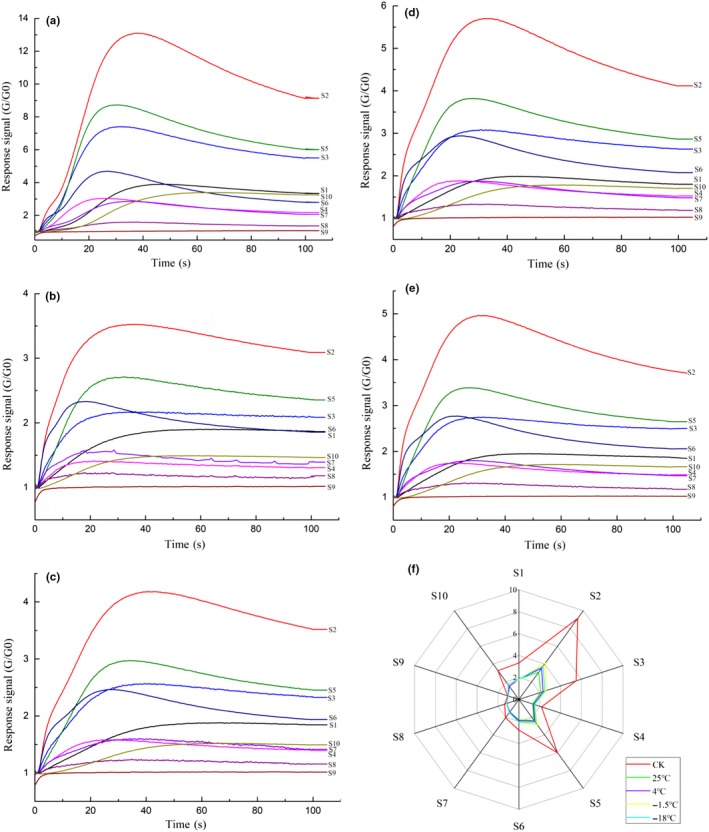
E‐nose response signals of NFC apple juices. (a) the freshly squeezed apple juice (control); (b) the juice stored at 25°C; (c) the juice stored at 4°C; (d) the juice stored at −1.5°C; and (e) the juice stored at −18°C, and the radar chart (f)

The e‐nose response signals of the juices including the control (the freshly squeezed apple juice) and the NFC juices stored at different temperatures are given in Figure 3. It was found that the response signals of sensor 2 (S2, the following abbreviations are the same), S5, and S3 in all the juices were significantly increased and then remained at a relatively high levels, indicating that the main aroma constituents in NFC apple juice may include ketones, aldehydes, and alkane organic and NO_X_ compounds. Besides, the response signals of the freshly squeezed apple juice were significantly higher than the juices stored for 150 days, suggesting that the aroma constituents in apple juice were decreased during the storage. Notably, the juice stored at −1.5°C showed the highest response levels compared with 25, 4, and −18°C, indicating a maximum retention of aroma. Additionally, the radar chart was yielded based on the average response signals of the 10 sensors during 100–105 s. As shown in Figure 3f, the different juice samples showed a similar shape but significantly different response strengths, which further confirmed the decrease in aroma constituents in NFC juice.

### 3.7 E‐tongue analysis of NFC juices

**Figure 4:**
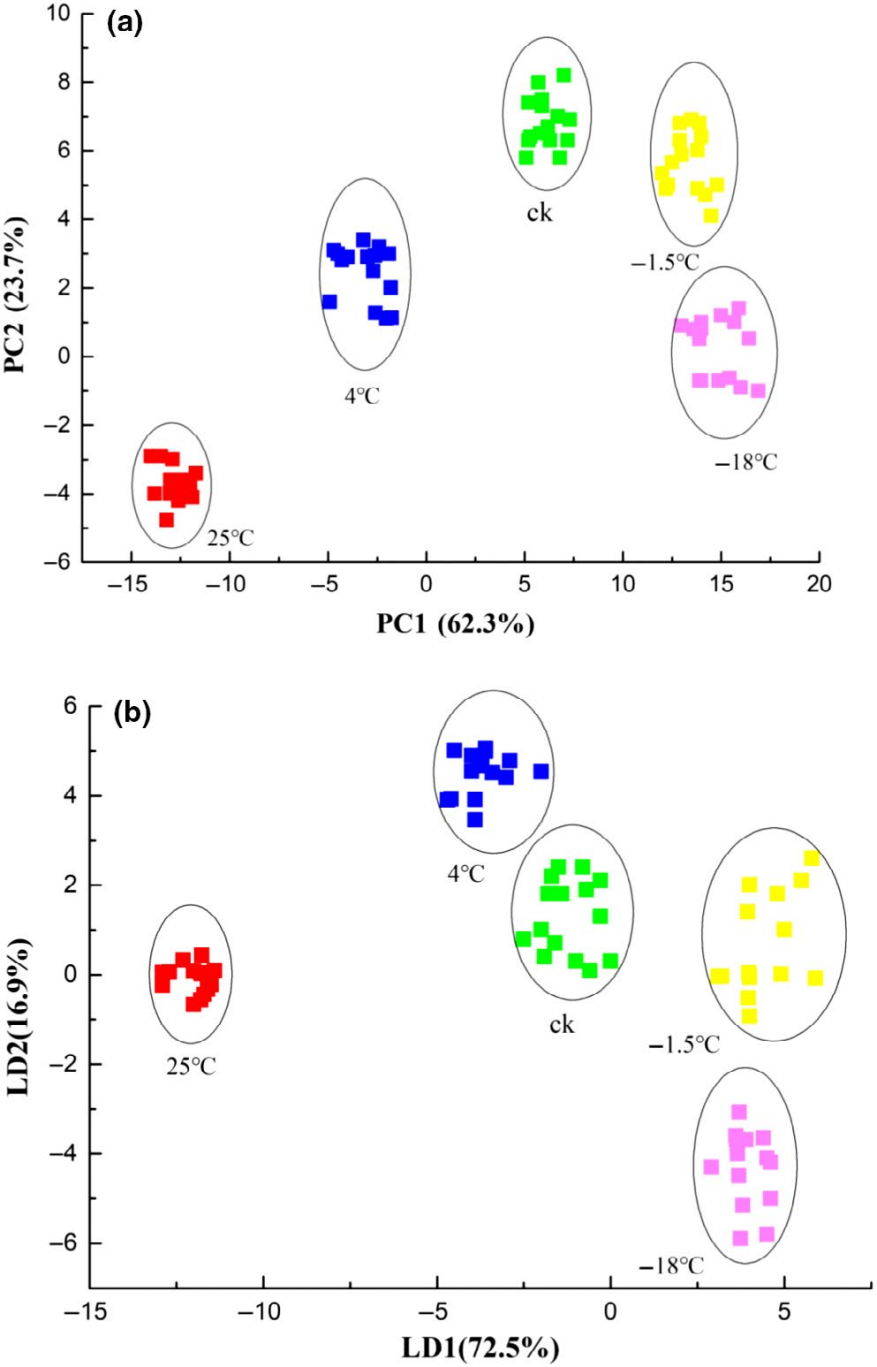
Electronic tongue‐based PCA (a) and LDA (b) score plots of the juices

The tastes of the juices were detected by e‐tongue, and the results were further analyzed by means of multivariate statistical analysis methods including PCA and LDA to pinpoint the possible alterations of the juices in taste. PCA is a classical unsupervised algorithm of pattern recognition to analyze, classify, and reduce the dimensionality of numerical data sets in a multivariate problem (Costache, Corcoran, & Puslecki, 2009). It can decompose the experimental data matrix into latent variables and explain the variables in data by two or three principal components, so as to elucidate the relationships between the original variables and their influence on the matrix (Tian, Deng, & Chen, 2007). LDA is a classical machine learning technique, which mainly uses the statistical information of image pixels while ignoring their scattering characteristics (Mahdianpari et al., 2018). As shown in Figure 4, all the juices were well separated into individual regions. Figure 4a suggests that the score plot of PCA explained 86% of the total variance, which consisted of PC1 (62.3%) and PC2 (23.7%). As a consequence, PC1 and PC2 can be considered adequate to represent the original data set. In general, the juice samples that can be grouped into a cluster were characterized by a high similarity (Tian et al., 2018). As shown in Figure 4a, the juices stored at different temperatures had significant difference in taste, whereas the juice stored at −1.5°C was found to have a more similar taste to the freshly squeezed apple juice.

To verify the result of PCA, LDA was performed to analyze the same data matrix. Figure 4b suggests that LD1 and LD2 could explain 72.5% and 16.9% of the total variance, respectively, and the combination of LD1 and LD2 made up to 89.4% of the original data information. Besides, it can be observed that LDA yielded a highly similar result to PCA. Both PCA and LDA suggested that the juice stored at −1.5°C exhibited a closer taste to the freshly squeezed juice. The results are consistent with a previous study by Guo et al. (2008), who reported that compared with room temperature and low temperature (4°C), controlled freezing point storage showed preferably higher taste qualities. However, exceedingly low temperature (i.e., −18°C) may also cause significant changes in the taste of the juices, as shown in Figure 4b. It may be due to the fact that the process of freezing and thawing would affect the texture of the pulp cells.

## 4 CONCLUSION

In this work, the NFC juices were prepared and then stored at a wide temperature range, as represented by 25, 4, −1.5, and −18°C, respectively. Through the detection of microorganisms, it was found that the low storage temperatures were positively related to the decreased growth rate of the yeast and mold. After 150 days of storage, both the aerobic plate counts and the yeast and mold counts were lower than the standard defined by Institute of Food Science and Technology (IFST), demonstrating good safety during the storage. Besides, compared with 4 and 25°C, the freezing point temperature (−1.5°C) could retard the decrease of the physicochemical properties and polyphenol contents. Also, the low temperature was favorable for maintaining the original color of the juice. However, an exceedingly low temperature such as −18°C could cause the significant loss of aroma and taste in NFC apple juice. The further PCA and LDA suggested that after 150 days of storage, the juice stored at −1.5°C exhibited the highest similarity to the freshly squeezed apple juice, which may indicate that −1.5°C was more favorable for storing NFC apple juice.

## CONFLICT OF INTERESTS

The authors declared no potential conflicts of interest with respect to the research, authorship, and/or publication of this article.

## ETHICAL STATEMENT

This study does not involve any human or animal testing.
