# Supplementary figures and images for: Freezing point temperature is in favor of not‐from‐concentrate apple juice storage
Source: Food Sci Nutr. 2019 May 29;7(7):2242–51. doi: 10.1002/fsn3.1028 (PMC6657751; doi:10.1002/fsn3.1028)

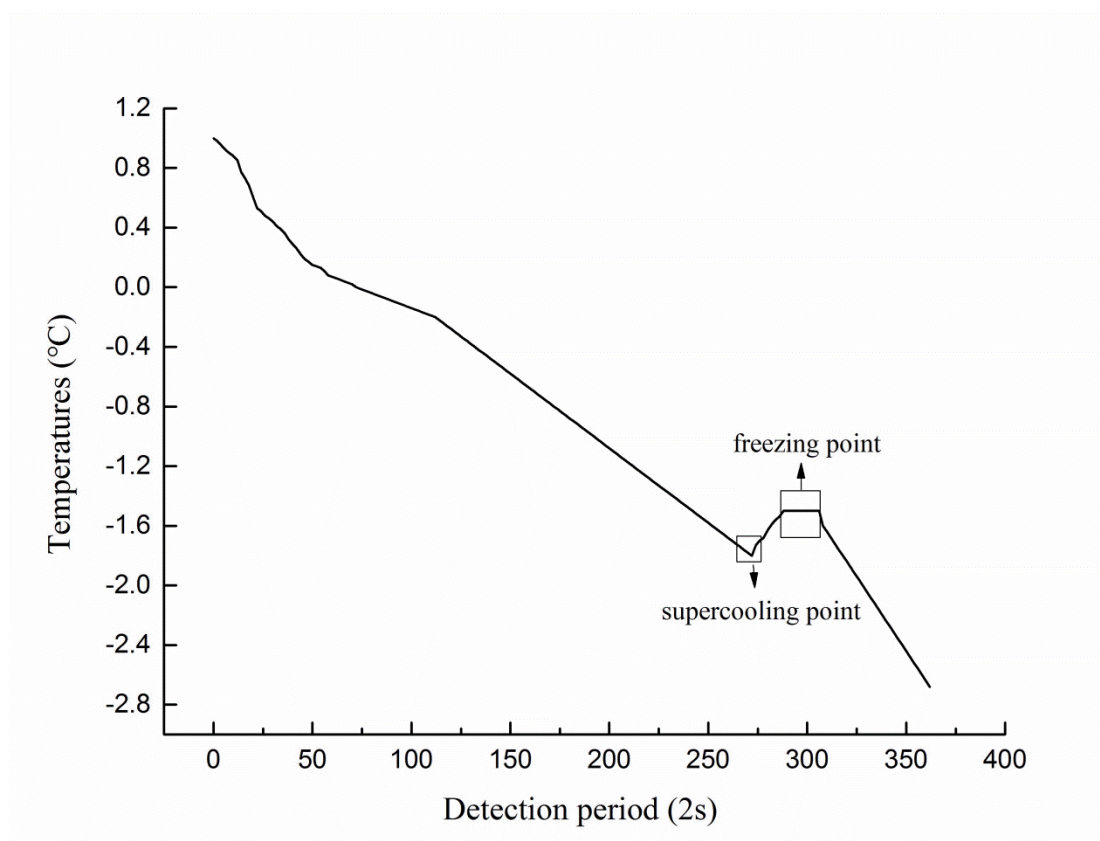

**Figure S1.** The temperature decreasing curve of not from concentrate apple juice.

Supplement: Supplementary file 1 [file FSN3-7-2242-s001.pdf]
