# Supplementary material for: Freezing point temperature is in favor of not‐from‐concentrate apple juice storage
Source: Food Sci Nutr. 2019 May 29;7(7):2242–51. doi: 10.1002/fsn3.1028 (PMC6657751; doi:10.1002/fsn3.1028)
Supplement: Supplementary file 2 [file FSN3-7-2242-s002.docx]

**Table S1.**Sensors used in e-nose and the main applications.

| Number | Name | Relative volatile compounds | Reference |
| --- | --- | --- | --- |
| S1 | W1C | Aromatic compounds | Toluene, 10 mg/L |
| S2 | W5S | Polar and nitrogen oxides | NO_2_, 1 mg/L |
| S3 | W3C | Aromatic compounds, ketones, and aldehydes | Benzene, 10 mg/L |
| S4 | W6S | Hydrogen | H_2_, 100 mg/L |
| S5 | W5C | Low polarity aromatic compounds and alkane | Propane, 1 mg/L |
| S6 | W1S | Broad-methane | CH_3_, 100 mg/L |
| S7 | W1W | Sulfur organic compounds and terpenes | H_2_S, 1 mg/L |
| S8 | W2S | Broad alcohols, ketones, and partially aromatic compounds | CO, 100 mg/L |
| S9 | W2W | Sulfur and aromatic compounds | H_2_S, 1 mg/L |
| S10 | W3S | Methane-aliphatic compounds | CH_3_, 100 mg/L |
